# Computed tomography radiomics of intratumoral and peritumoral microenvironments for identifying the invasiveness of subcentimeter lung adenocarcinomas

**DOI:** 10.1186/s12880-025-01882-z

**Published:** 2025-08-18

**Authors:** Yu-Qiang Zuo, Qing Liu, Tie-Zhi Li, Zhi-Hong Gao, Xu Yang, Yu-Ling Yin, Ping-Yong Feng, Zuo-Jun Geng

**Affiliations:** 1 Department of Physical Examination Center, The 2nd Hospital of Hebei Medical University, 215#, Heping West Road, Xinhua District, Shijiazhuang, Hebei 050000 People’s Republic of China; 2 Department of Imaging Center, The 2nd Hospital of Hebei Medical University, 215#, Heping West Road, Xinhua District, Shijiazhuang, Hebei 050000 People’s Republic of China; 3 Department of Thoracic Surgery, The 2nd Hospital of Hebei Medical University, 215#, Heping West Road, Xinhua District, Shijiazhuang, Hebei 050000 People’s Republic of China

**Keywords:** Lung adenocarcinoma, Subcentimeter, Radiomics, Invasiveness

## Abstract

**Background:**

The invasiveness of nodules plays a crucial role in the management and surgical methods selection of lung adenocarcinoma (LAC); however, the ability of traditional chest computed tomography (CT) imaging to detect the invasiveness of subcentimeter LAC is limited.

**Objective:**

Development and validation of a model based on computed tomography (CT) radiomics of the intratumoral and peritumoral microenvironments were used to identify the invasiveness of lung adenocarcinomas (LACs) appearing as subcentimeter nodules.

**Methods:**

A total of 142 consecutive patients with 142 pathologically confirmed subcentimeter LAC nodules were retrospectively studied from January 2020 to December 2023. The demographic data, clinical data, and CT features were retrospectively collected. A total of 2,264 radiomic features were extracted from LAC nodules in the intratumoral and peritumoral microenvironment and then used to construct the radiomic signature with the correlation coefficient and the least absolute shrinkage and selection operator (LASSO) logistic regression and generated radiomic scores (Radscores). A predictive model was constructed based on independent factors selected using a multiple logistic regression model. The performance of the model was evaluated with respect to its discrimination, calibration, and clinical utility.

**Results:**

In a total 142 LAC nodules, including 53 microinvasive adenocarcinoma (MIA) nodules and 89 invasive adenocarcinoma (IAC) nodules, the maximum diameter of nodules in the IAC group was larger than that of the MIA group. The positive rate of the vessel convergence sign (VCS) and vacuole sign in the IAC group were higher than that of the MIA group showing a statistical difference (*p* < 0.05). Logistic regression analysis showed that the maximum diameters of nodules and VCS were independent factors of IAC, but the predictive model based on CT features (maximum diameter and VCS) had moderate discriminative ability (area under the curve = 0.72), insufficient for standalone clinical use. The Radscores based on gross tumor volume (GTV), gross peritumoral volume (GPTV), and gross peritumoral region (GPR) in the IAC group were significantly higher than those of the MIA group (all *P* < 0.05, Mann-Whitney U test). The predictive model based on Radscores demonstrated improved discriminative ability (AUCs > 0.75) and calibration compared to CT features, though their clinical utility requires further validation.

**Conclusions:**

The CT features-based predictive model had limited ability to differentiate the invasiveness in subcentimeter LAC nodules. Models using GTV, GPTV, and GPR Radscores showed improved performance for predicting invasiveness, though further validation is needed, with the GTV-based model performing best. However, this study has limitations, including its retrospective single-center design and potential selection bias due to the small size of subcentimeter lung adenocarcinoma cases.

**Clinical trial number:**

Not applicable.

**Supplementary Information:**

The online version contains supplementary material available at 10.1186/s12880-025-01882-z.

## Key points

- A model based on computed tomography features had poor discriminative ability for assessing invasiveness in subcentimeter lung adenocarcinoma (LAC) lesions.
- A model based on radiomic features showed improved discriminative ability in predicting invasiveness of subcentimeter LAC lesions.
- The model based on gross tumor volume radiomic features reflected intratumoral heterogeneity and had excellent discriminative ability.
- Radiomic features of the peritumoral microenvironment had value in predicting the invasiveness of subcentimeter LAC lesions.

**Supplementary Information:**

The online version contains supplementary material available at 10.1186/s12880-025-01882-z.

## Background

With the popularization of computed tomography (CT) screening for lung cancer, frequently reported incidences of small-sized adenocarcinomas are increasingly reported in routine clinical practice [1]. Previous screening programs have shown that 60–70% of detected lung cancers were in stage I, and 56% were subcentimeter (lesions diameters < 1.0 cm) nodules [2]. Because of their small size, subcentimeter adenocarcinomas are often thought to be early-stage malignancies and less invasive [3]. However, malignant subcentimeter lung adenocarcinomas (LACs), can present with lymph node and distant metastases, and lymphatic, vascular, and pleural invasion are also more likely to occur in patients with solid subcentimeter LACs [4, 5], necessitating precise invasiveness assessment.

CT is the main noninvasive method to evaluate LACs. However, differential diagnosis of subcentimeter LACs is particularly difficult in clinical practice. The preoperative assessment of invasiveness in subcentimeter LACs presents significant challenges due to the frequent absence of typical CT imaging features (e.g., lobulation or spiculation) during this early stage combined with technical difficulty, low sensitivity, and limited diagnostic accuracy of invasive techniques like bronchoscopy or biopsy targeting these small nodules [6, 7]. For instance, Wu F et al. [8] reported AUCs of 0.622–0.641 for IACs vs. pre-IACs for traditional CT features in subcentimeter pure ground-glass nodules. These limitations are exacerbated in MIA/IAC lesions, where morphological changes are often subtle.

Radiomics, involving feature extraction, dimensionality reduction, and model construction, can obtain extensive mineable data hidden in CT images. It has proven to be a useful tool for identifying tumor heterogeneity and the microenvironment and can be used as predictive factors for tumor staging, differentiation, and prognosis [9–11]. Recent studies have demonstrated that radiomics can capture subvisual texture patterns and peritumoral alterations that are invisible to conventional CT assessment [7]. Specifically, the inclusion of peritumoral radiomic features was hypothesized to improve invasiveness prediction based on two key biological premises: (1) IACs exhibit microscopic stromal invasion beyond tumor margins, altering peritumoral tissue architecture [12, 13], and (2) peritumoral microenvironment changes (e.g., fibrotic response, angiogenesis) may precede macroscopic invasion [13]– [14]. Cho et al. [12] showed that marginal radiomics features could predict pathological invasion in LAC, while Lambrechts et al. [15] highlighted stromal remodeling as a hallmark of invasive tumors. However, the incremental value of peritumoral features remains debated, with studies like Wu et al. [16] reporting limited improvement over intratumoral features for small pulmonary nodules, underscoring the need for this investigation.

Given the difference in the extent of invasion into peritumoral tissue structures between MIA and IAC lesions, we hypothesize that radiomics of the peritumoral microenvironment can help to distinguish MIA lesions from IAC lesions. This study aims to systematically evaluate both intratumoral and peritumoral radiomic features to establish their respective and combined values in predicting invasiveness of subcentimeter LACs, addressing current gaps in CT-based assessment.

## Materials and methods

### Patients

This study was approved by the institutional review board and Ethical Committee of our institution, (approval No.: 2023-R384), which waived the requirement for patients’ informed consent of this retrospective study. The study adhered to the principles in the Declaration of Helsinki, and all information identifying patients was removed from the database.

**Fig. 1:**
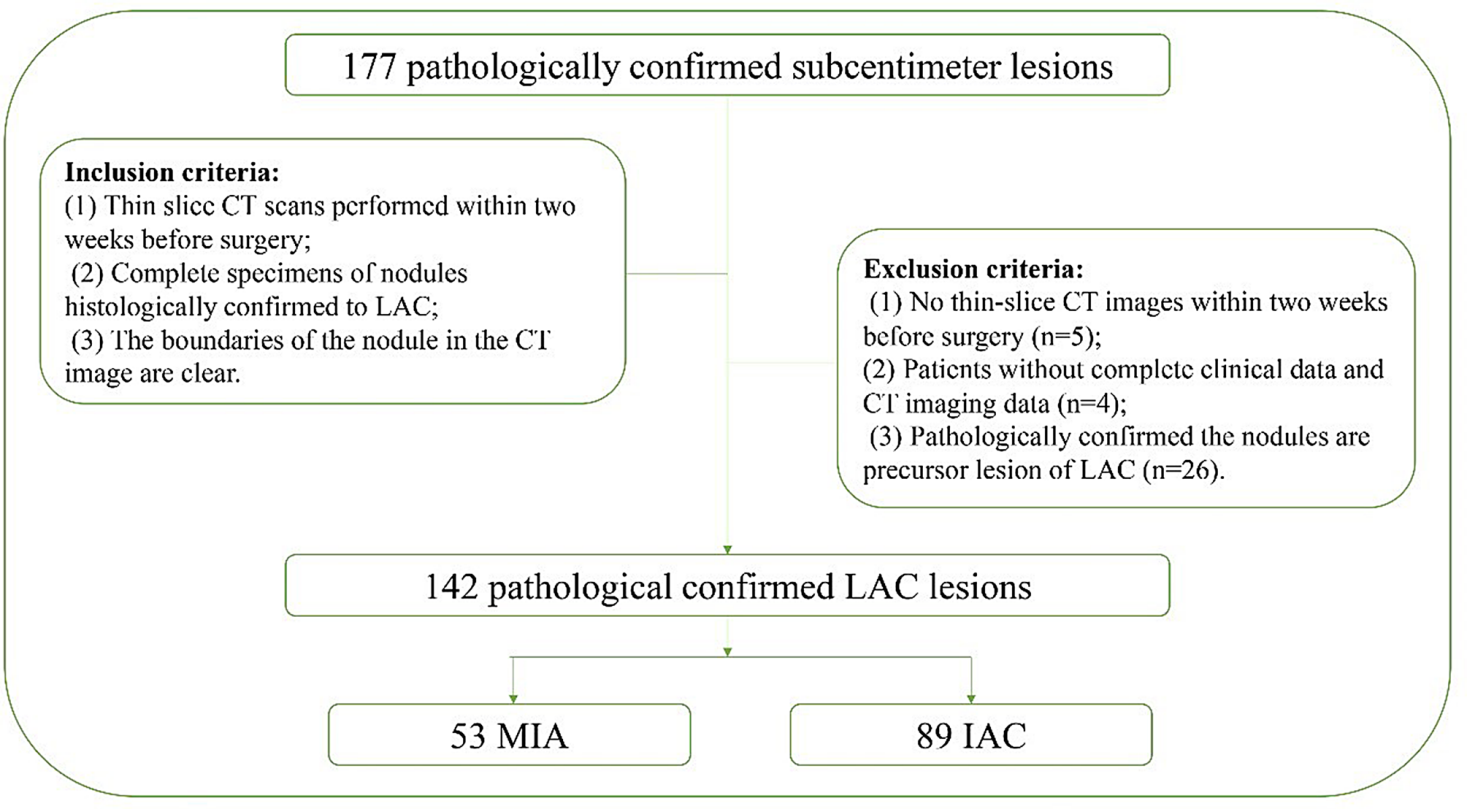
The flow chart of patient enrollment requirements

We retrospectively reviewed 142 patients with 142 subcentimeter nodules between January 2020 − December 2023, who were pathologically confirmed after thoracic surgical resection in our hospital. Inclusion criteria included the following: (1) thin slice CT (slice thickness < 1.5 mm) scans performed within 2 weeks before thoracic surgery, (2) complete specimens of subcentimeter nodules histologically confirmed to be MIA or IAC, and (3) clear boundaries of the nodules in the CT image, which allowed for precise delineation of the nodule’s boundaries. Exclusion criteria included the following: (1) no thin slice CT images within 2 weeks before surgery, (2) patients without complete clinical data and CT imaging data, and (3) pathological confirmation that the nodules were precursor lesions of LACs. Demographic data such as age, sex, height, weight, smoking status, drinking status, and family history of tumors were recorded. The flow chart of patient enrollment is shown in Fig. 1.

### CT examinations

All patients underwent a chest CT scan within 2 weeks before surgery, while in a supine position with their hands raised above their heads. Scans were from the thoracic entrance to the costophrenic angle. All patients underwent spiral CT scans after taking deep inspiratory breaths. The scanning equipment included a GE Optima660 64-slice spiral CT and Philips iCT 256-slice spiral CT. Scanning parameters included the following: tube voltage, 120 kVP; tube current, 100–300 mA. Adaptive tube current modulation technology was used during scanning, scanning matrix, 512 × 512; slice thickness, 5.0 mm; and spacing, 5.0 mm. The reconstructed slice was 1.0–1.25 mm thick. Lung window settings were the following: window width, 1,500 HU; window level, − 600 HU; using a mediastinal window setting (window width, 350 HU; window level, 40 HU).

### Collection of patient demographic data, CT features, and pathological data

Patient demographic data, CT features, and pathological data were collected, including sex, age, height, weight, family history of tumors, and smoking and drinking status. Body mass index (BMI) was calculated based on height and weight, using the formula: BMI = weight (kg)/ [height (m)]^2^.

Two radiologists with 15 years of experience were blinded to the patients’ clinical information. They evaluated the tumor CT features [tumor maximum diameter, tumor border (clear, unclear), location (right upper lobe, right middle lobe, right lower lobe, left upper lobe, and left lower lobe), lobulation sign, spiculation sign, vessel convergence sign (VCS), pleural indentation sign, vacuole sign, and air bronchogram sign] using picture archiving and a communication system (PACS). Discrepancies in interpretations between radiologists were resolved by consensus. A pathologist with more than 10 years of experience recorded the pathological results, diagnosed according to the 2021 WHO classification of lung tumors [17].

**Fig. 2:**
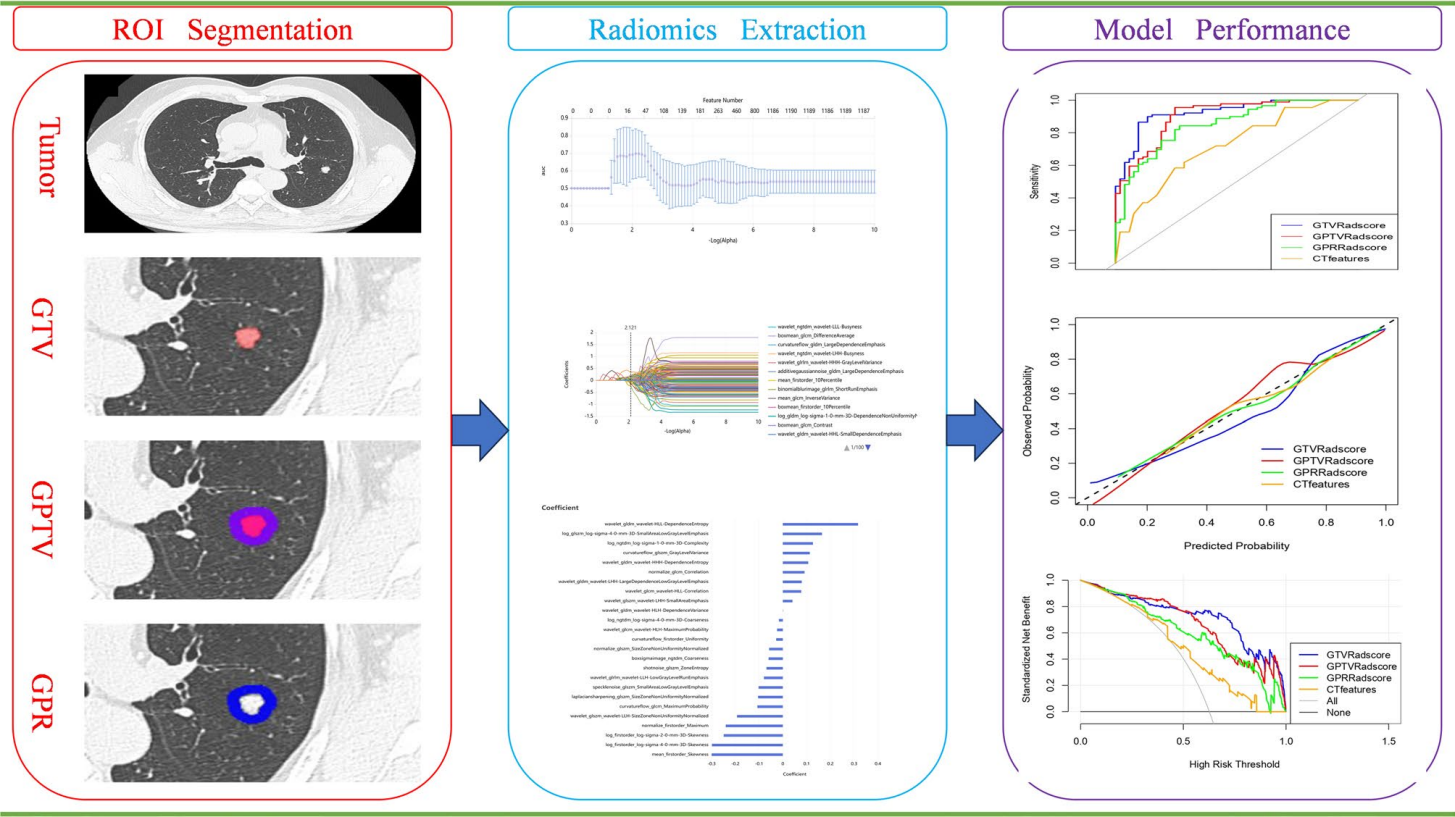
The workflow of the subcentimeter lung adenocarcinomas nodules segmentation, radiomic extraction, model construction, and validation

Workflow details are shown in Fig. 2, including regions of interest (ROIs) of LAC segmentation, radiomic feature extraction, feature selection, and construction of predictive models using the uAI Research Portal V1.1 (Shanghai United Imaging Intelligence, Shanghai, China) [18]. 

### ROIs segmentation, radiomic features extraction, and selection

Initially, all thin slice CT images with lung windows using Digital Imaging and Communications in Medicine format were transferred and stored. Subsequently, they were imported into software of the uAI (United Imaging Intelligence) research portal, which is an open-source image processing software [18]. The software automatically identified and segmented all pulmonary nodules, which were all in three dimensions. All auto-segmented contours were then manually reviewed and refined by two radiologists (each with > 10 years of experience) to exclude adjacent non-tumoral structures (e.g., vessels, bronchi, chest wall) and ensure boundary accuracy. The gross tumor volume (GTV) was defined as the whole tumor area. Using the software, we dilated GTV outward by 5 mm, creating ROIs for gross peritumoral tumor volumes (GPTV), based on the pathological evidence of microscopic tumor extension for adenocarcinoma [19]. ROI’s reliability was verified based on prior validation of identical protocols [18]. The spherical structure formed by dilating the tumor boundary by 5 mm was called the gross peritumoral region (GPR). Before radiomics extraction, we used the following measurements to eliminate heterogeneity between different scanners. First, grey intensity normalization and resampling were performed, and the images were normalized by subtracting the window level (− 600) and dividing by the window width (1,500), then resamplinjg to 1 × 1 × 1 mm^3^ voxels using the B-Spline interpolation method. The binWidth was set to 25 for discretization of the image gray level. The radiomic features were then extracted from the ROIs, including first-order, shape, grey-level co-occurrence matrix, grey-level run-length matrix, grey-level size zone matrix features, grey-level dependence matrix, and neighboring grey size zone matrix. A total of 2,264 features were extracted for each ROI. The intraclass correlation coefficient method was used to guarantee the reliability and repeatability of radiomics features, then the minimum-redundancy maximum-relevance (mRMR) method was used to eliminate the redundant and irrelevant radiomics features. LASSO with 10-fold cross-validation was conducted to reduce feature dimensionality. This internal validation approach was prioritized over external validation due to cohort size constraints, ensuring rigorous evaluation while maximizing statistical power. The optimal radiomic features selected from candidate features were used to construct radiomic models, then radiomic scores (Radscores) were calculated.

### Construction and performance of the predictive model

First, we constructed IAC prediction models using demographic data, CT features, and the Radscores of GTV, GTRV, and GPR. To avoid multicollinearity between radiomic features and CT morphological characteristics, we intentionally isolated the predictors in separate modeling frameworks: CT feature model: exclusively used traditional features (maximum diameter and VCS); Radiomics models: solely employed Radscores (GTV, GPTV and GPR) .Then, we comprehensively evaluated the performance of these prediction models using a receiver operating characteristic (ROC) curve, Hosmer-Lemeshow test, and decision curve analysis (DCA).

### Statistical analysis

Data processing and modeling were performed using R (version 4.1.3, www.R-project.org). The Shapiro-Wilk test assessed continuous variable normality. The Kolmogorov-Smirnov test was used to identify parametric distributions. The *t*-tests were used for parametric data, and expressed as the mean ± standard deviation (x̅ ± s), and the Mann-Whitney U test was used for nonparametric data, expressed as the median and quartile (M, IQR). The chi-squared test or Fisher’s quartile test was used for semantic signs. For comparisons of categorical variables, the chi-square (χ2) test or Fisher’s exact test was used. Univariable and multivariable logistic regression analyses identified independent predictors for IAC in LAC patients. The ROC curve was drawn, and the area under the curve (AUC) was calculated to evaluate diagnostic performance. 95% confidence intervals (CI) for AUC values were calculated using DeLong’s nonparametric method with 2000 bootstrap iterations. Calibration curves accompanied by the Hosmer-Lemeshow test were plotted to evaluate predictive accuracies of the models. DCA was conducted to evaluate whether the models were sufficiently robust. Significance using the DeLong test suggested differences and stability between models. A bilateral value of *p* < 0.05 was considered statistically significant.

## Results

### Clinical characteristics and CT features, and the construction and performance of the predictive model based on CT features

**Fig. 3:**
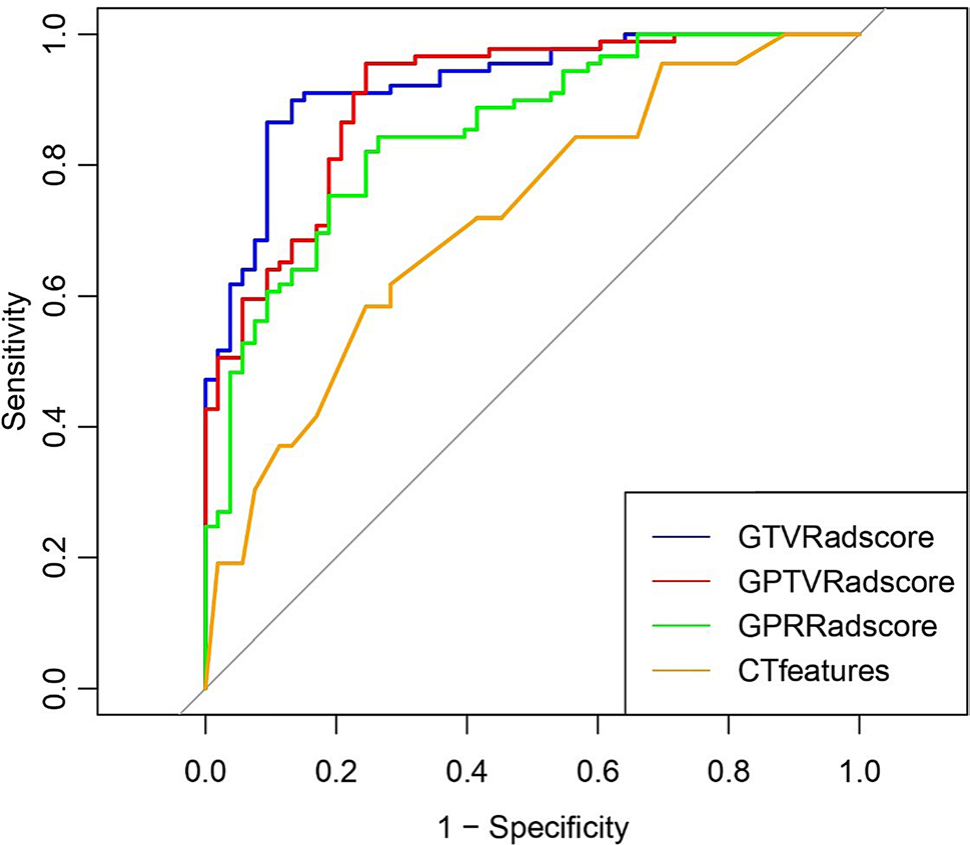
Receiver operating curve analysis of the predictive models

This study retrospectively included 142 patients with a total of 142 subcentimeter LAC nodules, including 53 MIA nodules and 89 IAC lesions. The comparison of demographic data and CT features between the MIA and IAC groups is shown in Table 1. Compared with the MIA group, the IAC group had a larger maximum diameter, higher rate of VCS and vacuole sign, and differences between the MIA and IAC groups were statistically significant (*p* < 0.05). However, there were no statistically significant difference between the MIA and IAC groups in terms of sex, age, BMI, family history of tumors, smoking status, drinking status, nodule location, tumor border, lobulation sign, speculation sign, pleural indentation sign, or air bronchogram sign (*p* > 0.05) (Table 1). Multivariate logistic regression analysis showed that the VCS [odds ratio (OR): 3.002, 95% confidence interval (CI):1.263–7.13; *p* = 0.013, *p* < 0.05] and max diameter (OR: 1.441, 95% CI: 1.109–1.873; *p* = 0.006, *p* < 0.05) were independent factors for the IAC (Table 2), therefore we used the VCS and max diameter to construct the predictive model. Regardless, however, the AUC was poor (AUC < 0.75), even if we used the combined prediction probability of the maximum diameter and VCS (Table 3; Fig. 3).

**Table 1:** Demographic data and computed tomography features of the MIA and IAC groups

Characteristics	MIA(*n* = 53)	IAC(*n* = 89)	t/u/χ^2^	*p* value
Age	52.00(18.50)	57.00(16.00)	-1.794u	0.073
Gender			0.057a	0.811
Female	42(79.25%)	69(77.53%)		
Male	11(20.75%)	20(22.47%)		
BMI	24.80(5.27)	25.39(4.87)	-0.976u	0.329
Max Diameter	8.00(2.65)	9.00(2.00)	-3.583u	< 0.001
LAC CT image Type			0.309a	0.578
Subsolid	47(88.68%)	76(85.39%)		
Solid	6(11.32%)	13(14.61%)		
Family history of tumor			0.150b	0.699
No	49(92.45%)	85(95.51%)		
Yes	4(7.55%)	4(4.49%)		
Smoking			0.447a	0.504
No	45(84.91%)	79(88.76%)		
Yes	8(15.09%)	10(11.24%)		
Drinking			0.045b	0.832
No	49(92.45%)	80(89.89%)		
Yes	4(7.55%)	9(10.11%)		
Location			4.994c	0.286
RUL	20(37.74%)	23(25.84%)		
RML	3(5.66%)	10(11.24%)		
RLL	14(26.42%)	17(19.10%)		
LUL	9(16.98%)	20(22.47%)		
LLL	7(13.20%)	19(21.35%)		
border			1.182b	0.277
Clear	4(7.55%)	2(2.25%)		
Unclear	49(92.45%)	87(97.75%)		
Lobulation sign			0.275a	0.600
No	22(41.51%)	33(37.08%)		
Yes	31(50.49%)	56(62.92%)		
spiculation sign			0.030a	0.862
No	26(49.06%)	45(50.56%)		
Yes	27(50.94%)	44(49.44%)		
VCS			11.493a	0.001
0	44(83.02%)	49(55.06%)		
1	9(16.98%)	40(44.94%)		
Pleural indention sign			0.204a	0.652
No	33(62.26%)	52(58.43%)		
Yes	20(37.74%)	37(41.57%)		
vacuole sign			4.771a	0.029
No	46(86.79%)	63(70.79%)		
Yes	7(13.21%)	26(29.21%)		
Air bronchogram sign			1.654b	0.198
No	53(100.00%)	84(94.38%)		
Yes	0(0.00%)	5(5.62%)		

MIA, microinvasive adenocarcinoma; IAC, invasive adenocarcinoma; LAC, lung adenocarcinoma; VCS, vascular converge sign; RUL, right upper lobe; RML, right middle lobe; RLL, right lower lobe; LUL, left upper lobe; LLL, left lower lobe

**Table 2:** Univariate and multivariate analysis of factors differentiating between the MIA and IAC groups, presented as subcentimeter lesions

Characteristics	Univariate LR analysis		Binary multivariate LR analysis	
	OR (95%CI)	*p* value	OR (95%CI)	*p* value
Max Diameter	1.569(1.221～2.015)	0.000	1.441(1.109～1.873)	0.006
VCS	3.991(1.741～9.151)	0.001	3.002(1.263～7.131)	0.013
vacuole sign	2.712(1.084～6.785)	0.033	1.881(0.704～5.025)	0.208

LR, logistic regression; OR, odds ratio; VCS, vascular converge sign

**Table 3:** The receiver operating curve performance of computed tomography features to predict the IAC

Characteristics	AUC(95%CI)	Sensitivity	Specificity	Cut-off value	*p* value
Max Diameter	0.677(0.587～0.767)	36.0%	91.06%	9.80	< 0.001
VCS	0.640(0.548～0.732)	44.9%	83.0%		0.005
CT features	0.720(0.635～0.806)	58.4%	75.5%	0.69	< 0.001

AUC, area under curve; VCS, vascular converge sign; CT, computed tomography

Computed tomography features: Combined prediction probability of the maximum diameter and VCS

### Radscores of GTV, GPTV, and GPR and the construction and performance of the predictive model based on radscores

By using correlation coefficient analysis and LASSO to reduce the dimensions of features, 25, 28, and 13 radiomic features were selected from the initial 2,264 features for GTV, GPTV, and GPR, respectively. We then used these features to select and build a radiomics score calculation formula. The formulas for the GTV, GPTV, and GPR Radscores are shown in Figure E1. Compared to the MIA group, the IAC group had significantly higher GTV, GPTV, and GPR Radscores (all, *p* < 0.05; Table 4). We then used the GTV, GPTV, and GPR Radscores to construct a predictive model. Model performance was evaluated via 10-fold cross-validation to mitigate overfitting risk inherent in single-center studies with limited samples. ROC curves showed that all Radscores had good performance (AUC > 0.75) and good sensitivity and specificity (Table 5; Fig. 3).

**Table 4:** Radscores of MIA group and IAC group

Characteristics	MIA(*n* = 53)	IAC(*n* = 89)	t	*p* value
GTV Radscore	0.40 ± 0.18	0.77 ± 0.18	-11.734	< 0.001
GPTV Radscore	0.44 ± 0.18	0.76 ± 0.17	-10.587	< 0.001
GPR Radscore	0.52 ± 0.12	0.70 ± 0.13	-8.156	< 0.001

GTV, gross tumor volume; GPTV, gross peritumoral tumor volume; GPR, gross peritumoral region; MIA, microinvasive adenocarcinoma; IAC, invasive adenocarcinoma

**Table 5:** The receiver operating curve performance of the GTV, GPTV, and GPR radscores for predicting the IAC

Characteristics	AUC	95%CI	Sensitivity	Specificity	Cut-Off value	*p* value
GTV Radscore	0.926	0.883～0.968	86.5%	90.6%	0.61	< 0.001
GPTV Radscore	0.904	0.854～0.955	95.5%	75.5%	0.51	< 0.001
GPR Radscore	0.852	0.789～0.915	84.3%	73.6%	0.59	< 0.001

GTV, gross tumor volume; GPTV, gross peritumoral tumor volume; GPR, gross peritumoral region, AUC, area under curve

### Predictive model comparisons and validations

**Fig. 4:**
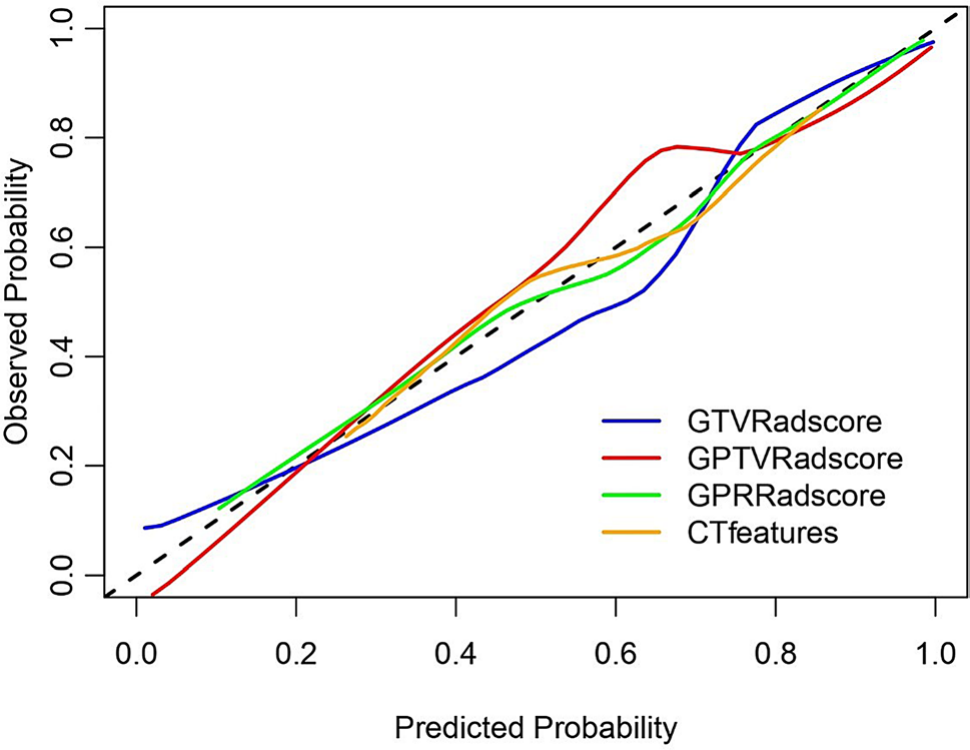
Calibration of different models

**Fig. 5:**
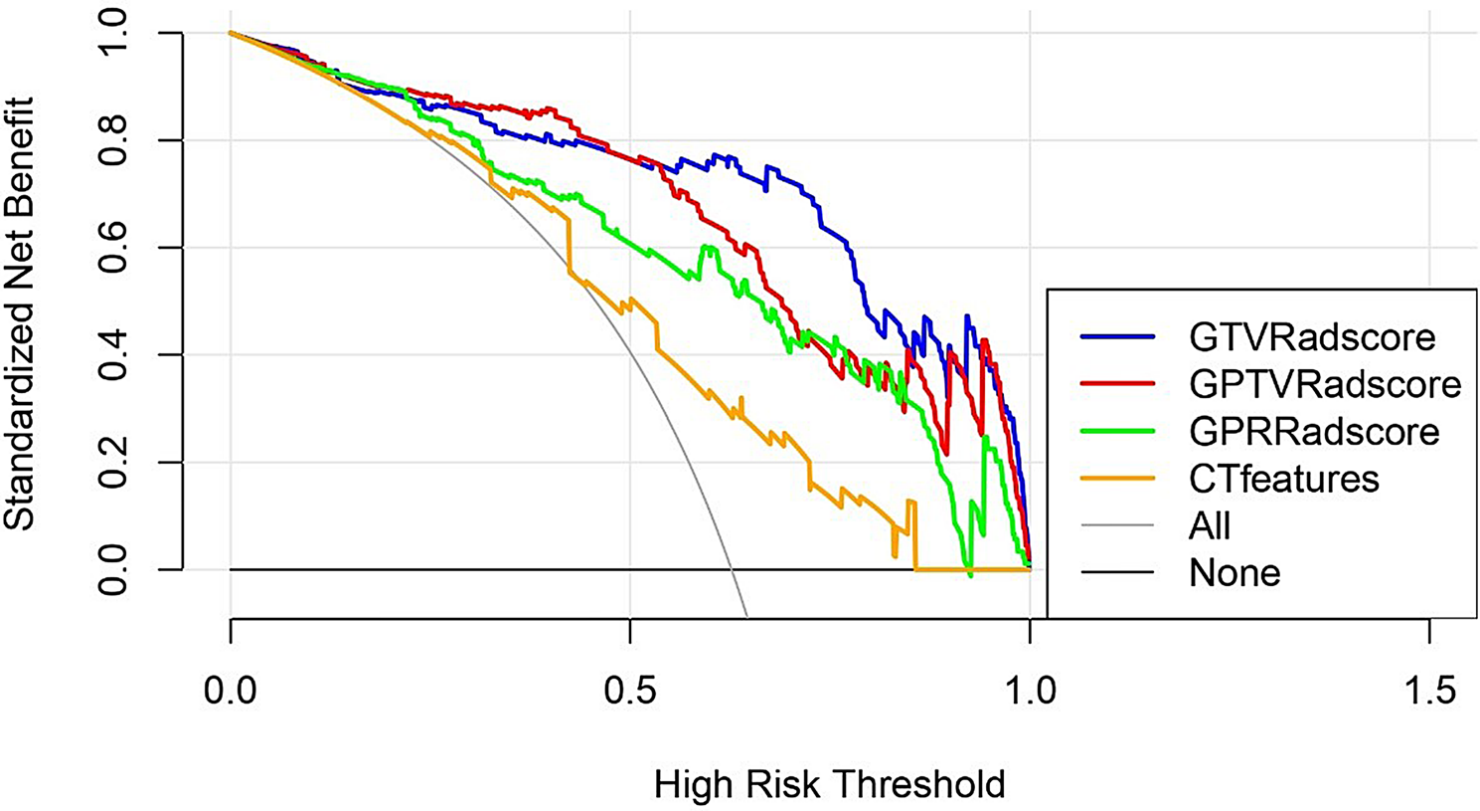
Decision curve analysis of different models

DeLong’s test was used to compare the AUC of different predictive models. The results showed that the AUCs of the GTV, GPTV, and GPR Radscores were all higher than the CT features. Furthermore, the AUCs of GTV and GPTV Radscores were statistically greater than the AUC of the GPR Radscore (all, *p* < 0.05; Table 6). The predictive models constructed using the Radscores all had good fitness and clinical utilities, with the predictive model constructed using the GTV Radscore having the best performance (Tables 5 and 6; Figs. 4 and 5). Threshold probabilities for DCA were defined through ROC optimization: GTV Radscore (0.136-1.000), GPTV Radscore (0.001-1.000), GPR Radscore (0.011–0.923), and CT features (0.220–0.424, 0.437–0.857). Clinically optimal thresholds (e.g.,0.61 for GTV Radscore) were identified where sensitivity and specificity converged.

**Table 6:** Comparisons of different predictive models

Models	Z value	*p* value
GTV Radscore vs. CT features	4.449	< 0.0001
GPTV Radscore vs. CT features	3.804	0.0001
GPR Radscore vs. CT features	2.621	0.0088
GTV Radscore vs. GPR Radscore	2.521	0.0117
GPTV Radscore vs. GPR Radscore	2.186	0.0288
GTV Radscore vs. GPTV Radscore	0.844	0.3987

GTV, gross tumor volume; GPTV, gross peritumoral tumor volume; GPR, gross peritumoral region; CT, computed tomography

## Discussion

The aim of this study was to determine the accuracy of CT features and radiomic features in predicting IAC in subcentimeter LAC lesions. The results showed that the predictive model based on CT features showed poor performance (AUC < 0.75, with low sensitivity). We then generated the Radscore based on GTV, GPTV, and GPR radiomic features, which showed a significant difference between the MIA and IAC cohorts, with the model based on radiomics having good performance and the model based on the GTV Radscore having the best performance (AUC > 0.75, with high sensitivity and specificity).

CT is the most commonly used technique for the detection and differentiation of LAC invasiveness [20]. Current guidelines largely consider size and shape characteristics as predictive of aggressiveness [21]. In the present study, the LAC lesion maximum diameter and the VCS were independent factors of the IAC. Larger tumor size is generally associated with higher invasiveness and worse prognoses, which has been well-documented in past reports [22–24]. VCS was also an independent risk factor of IAC, which was the same conclusion as previous studies [25, 26]. The reasons for these results may be the following. First, increased infiltration leads to higher oxygen consumption, affecting blood vessels by increasing their permeability and diameter. Second, a higher degree of invasion results in more fibrosis, which further causes blood vessels to aggregate around the tumor [25]. However, in this study, the predictive model based on tumor maximum diameter and VCS had poor discriminative ability and low sensitivity, which was consistent with the conclusions of previous studies [27–29]. Overall, these results further confirmed that CT features had limited value in distinguishing the invasiveness of subcentimeter LACs.

In the present study, we used radiomics based on GTV, GPTV, and GPR to construct a predictive model for subcentimeter LAC lesions. The results showed that a model based on GTV Radscores demonstrated the highest discriminative ability in this cohort because it had the highest discriminative efficiency, good fitness, and the best clinical utility. The model based on GPTV was the next best, followed by the model based on GPR radiomics, which was the worst. Although the model based on GPR was the worst, it still had good discriminative ability, calibration, and clinical utility. The reason for this phenomenon may be that the radiomic features of the peritumoral region reflected cancerous heterogeneity, and the crucial information could be indicative of changes in the areas surrounding tumors, such as biological aggressiveness, microinvasion, and micrometastasis [13]. However, the GTV-based model showed statistically superior performance to the GPR model (DeLong’s test, *P* = 0.012), while the GPTV-based model (conbining GTV and GPR features) exhibited intermediate efficacy. This result was consistent with the results of previous studies [14, 15], indicating that the radiomic features of the peritumoral microenvironment had clinical value in assessing the invasiveness of subcentimeter LACs, but could not improve the model performance. The underlying reason for the variability of Radscores may be due to the following: (1) Intraumoral heterogeneity drives imaging signatures: IAC exhibits highly heterogeneous cell populations within the tumor core (e.g., hyperproliferative clones, necrotic foci, fibrotic zones). These pathological features are directly quantifiable through CT texture analysis, reflecting heterogeneity in driver gene mutations (e.g., EGFR/KRAS) [30]; (2) Peritumoral noise compromises specificity: The mean peritumoral invasion extent of lung adenocarcinoma is about 2.9 mm [19], yet our 5-mm peritumoral region encompasses non-tumoral structures (vessels, bronchi, interstitial tissue). Radiomic features from these structures reduce specificity for IAC detection. In subcentimeter nodules, limited stromal remodeling (e.g., insufficient angiogenesis) combined with secondary changes (inflammation, edema) lowers the signal-to-noise ratio in GPTV features [31]. This explains GPTV’s high sensitivity (95.5%) but reduced specificity (75.5%). (3) Size constraints limit peritumoral utility: The peritumoral zone constitutes a smaller proportion in subcentimeter lesions, while pathological invasion extent remains limited (< 5 mm). Consequently, peritumoral features fail to capture early invasive signals before substantial microenvironmental alterations occur [3]. This radiomics model enhances clinical decision-making for indeterminate subcentimeter pulmonary nodules by providing quantifiable invasiveness probabilities (GTV Radscore > 0.61), enabling risk-stratified management where high-risk nodules (Radscore ≥ 0.61) may warrant video-assisted thoracoscopic surgery resection while low-risk cases (Radscore < 0.61) can undergo CT surveillance. Furthermore, it facilitates personalized surgical planning by guiding resection extent—sublobar resection for predicted MIA (lung preservation) versus lobar resection for IAC (oncological radicality). By integrating radiomic biomarkers with established guidelines for management of lung nodule of Fleischner society [7], the model improves diagnostic accuracy beyond conventional size and morphology assessment.

This study had several limitations. First, this retrospective single-institutional design, compounded by exclusive inclusion of surgically resected nodules may overrepresent aggressive/operable tumors, which carries an inherent risk of selection bias and limiting generalizability to surveillance-managed nodules. Second, due to the absence of an independent external cohort, despite rigorous internal cross-validation, future multi-center studies with larger samples are warranted to address these constraints. Third, this study included only surgically resection of histologically confirmed subcentimeter LAC lesions. So, in the selecting process, it was possible that lesion morphology had more malignant features. Finally, variations in CT acquisition parameters (e.g., scanner manufacturers, reconstructive kernels, tube current) could affect radiomic feature stability despite preprocessing normalization [32, 33]. This technical heterogeneity remains a confounder for multi-center reproducibility. Additionally, the exclusion of non-surgical nodules (e.g., surveillance-managed GGNs) further constrain generalizability to the broader spectrum of subcentimeter pulmonary lesions.

## Conclusion

The CT features-based predictive model had limited ability to differentiate the invasiveness in subcentimeter LAC nodules. While models based on GPTV and GPR Radscores (reflecting peritumoral microenvironment) showed value in predicting invasiveness, the model based on GTV Radscores (capturing intratumoral heterogeneity) showed superior discriminative in this study, but its clinical utility requires validation in larger multi-center cohorts. However, this study has limitations, including its retrospective single-center design and potential selection bias due to the small sample size of subcentimeter lung adenocarcinoma cases. Future prospective multi-center studies with larger cohorts are warranted to validate these findings.

## Supplementary Information

Below is the link to the electronic supplementary material.

Supplementary Material 1

## Data Availability

The datasets used and analysed during the current study are available from the first author or corresponding author on reasonable request.

## Abbreviations

BMI: Body mass index
CI: Confidence interval
CT: Computed Tomograph
DCA: Decision curve analysis
DICOM: Digital imaging and communications in medicine
GLCM: Grey-level co-occurrence matrix
GLDM: Grey-level dependence matrix
GLRLM: Grey-level run-length matrix
GLSZM: Grey-level size zone matrix features
HE: Hematoxylin and eosin
IASLC: International Association for the Study of Lung Cancer
LAC: Lung adenocarcinoma
LASSO: Least absolute shrinkage and selection operator
LR: Logistic regression
mRMR: Minimum-redundancy maximum-relevance
NGTDM: Neighbouring grey size zone matrix
NSCLC: Non-small cell lung cancer
OR: Odds ratio
PACS: Picture archiving and communication system
ROI: Region of interest
