# Supplementary material for: Computed tomography radiomics of intratumoral and peritumoral microenvironments for identifying the invasiveness of subcentimeter lung adenocarcinomas
Source: BMC Med Imaging. 2025 Aug 18;25:331. doi: 10.1186/s12880-025-01882-z (PMC12359731; doi:10.1186/s12880-025-01882-z)
Supplement: Supplementary file 1 — Supplementary Material 1 [file 12880_2025_1882_MOESM1_ESM.pdf]

GTV Radscore = 0.3165515 * wavelet_gldm_wavelet-HLL-DependenceEntropy + 0.164870724 * log_glszm_log-sigma-4-0-mm-3D-SmallAreaLowGrayLevelEmphasis + 0.126904041 * log_ngtdm_log-sigma-1-0-mm-3D-Complexity + 0.113473125 * curvatureflow_glszm_GrayLevelVariance + 0.106981568 * wavelet_gldm_wavelet-HHH-DependenceEntropy + 0.09141379 * normalize_glcm_Correlation + 0.07974526 * wavelet_gldm_wavelet-LHH-LargeDependenceLowGrayLevelEmphasis + 0.07758818 * wavelet_glcm_wavelet-HLL-Correlation + 0.0407067053 * wavelet_glszm_wavelet-LHH-SmallAreaEmphasis + 0.00104143191 * wavelet_gldm_wavelet-HLH-DependenceVariance + -0.0164767951 * log_ngtdm_log-sigma-4-0-mm-3D-Coarseness + -0.0240946654 * wavelet_glcm_wavelet-HLH-MaximumProbability + -0.0279087443 * curvatureflow_firstorder_Uniformity + -0.057350602 * normalize_glszm_SizeZoneNonUniformityNormalized + -0.0596029721 * boxsigmaimage_ngtdm_Coarseness + -0.06863495 * shotnoise_glszm_ZoneEntropy + -0.0795449838 * wavelet_glrlm_wavelet-LLH-LowGrayLevelRunEmphasis + -0.101834066 * specklenoise_glszm_SmallAreaLowGrayLevelEmphasis + -0.103941105 * laplaciansharpening_glszm_SizeZoneNonUniformityNormalized + -0.107107528 * curvatureflow_glcm_MaximumProbability + -0.1925526 * wavelet_glszm_wavelet-LLH-SizeZoneNonUniformityNormalized + -0.240177765 * normalize_firstorder_Maximum + -0.248686984 * log_firstorder_log-sigma-2-0-mm-3D-Skewness + -0.29812485 * log_firstorder_log-sigma-4-0-mm-3D-Skewness + -0.299564749*mean_firstorder_Skewness + 1.09561145

GPTV Radscore = 0.3507494 * shotnoise_glcm_Correlation + 0.273062617 * shotnoise_glcm_Imc2 + 0.199631259 * wavelet_glszm_wavelet-HLH-GrayLevelNonUniformityNormalized + 0.0817655846 * original_shape_Maximum2DDiameterSlice + 0.0744973645 * log_firstorder_log-sigma-4-0-mm-3D-InterquartileRange + 0.0526754819 * wavelet_glrlm_wavelet-HLH-LongRunLowGrayLevelEmphasis + 0.0418158025 * log_glszm_log-sigma-2-0-mm-3D-ZoneEntropy + 0.018020954 * normalize_firstorder_RootMeanSquared + 0.00498362 * wavelet_ngtdm_wavelet-HLH-Busyness + 0.004641537 * normalize_firstorder_90Percentile + -0.0122180227 * log_glszm_log-sigma-2-0-mm-3D-LowGrayLevelZoneEmphasis + -0.0123085957 * wavelet_glszm_wavelet-LLH-SizeZoneNonUniformityNormalized + -0.0144286864 * log_firstorder_log-sigma-0-5-mm-3D-Kurtosis + -0.014754829 * wavelet_ngtdm_wavelet-HHL-Coarseness + -0.015253596 * mean_glcm_Imc1 + -0.0248986483 * wavelet_firstorder_wavelet-LLH-Kurtosis + -0.0430043861 * specklenoise_glrlm_ShortRunLowGrayLevelEmphasis + -0.0532915257 * log_glszm_log-sigma-1-0-mm-3D-LowGrayLevelZoneEmphasis + -0.06455634 * log_ngtdm_log-sigma-2-0-mm-3D-Contrast + -0.066721566 * wavelet_glszm_wavelet-HHL-ZonePercentage + -0.06937302 * original_glcm_Idmn + -0.07130283 * normalize_firstorder_Maximum + -0.105942413 * log_firstorder_log-sigma-1-0-mm-3D-Kurtosis + -0.128801748 *

boxsigmaimage_gldm_SmallDependenceLowGrayLevelEmphasis + -0.132546946 * normalize_gldm_SmallDependenceHighGrayLevelEmphasis + -0.146063834 * laplaciansharpening_glcm_Idmn + -0.181797862 * log_firstorder_log-sigma-2-0-mm-3D-Minimum + -0.296858519*wavelet_gldm_wavelet-HHL-SmallDependenceLowGrayLevelEmphasis + 0.699660063

GPR Radscore = 0.204981625 * wavelet_glrlm_wavelet-LLL-RunLengthNonUniformity + 0.138715431 * boxsigmaimage_glszm_GrayLevelNonUniformityNormalized + 0.119720161 * normalize_glcm_Imc2 + 0.09172669 * original_shape_Maximum2DDiameterSlice + 0.056571 * log_glszm_log-sigma-0-5-mm-3D-SmallAreaEmphasis + -0.03993599 * log_glszm_log-sigma-4-0-mm-3D-LowGrayLevelZoneEmphasis + -0.0417297967 * recursivegaussian_glrlm_GrayLevelNonUniformityNormalized + -0.0443645865 * wavelet_gldm_wavelet-LLL-LowGrayLevelEmphasis + -0.06905209 * discretegaussian_glrlm_GrayLevelNonUniformityNormalized + -0.154182062 * wavelet_glszm_wavelet-LLH-SizeZoneNonUniformityNormalized + -0.1630785 * wavelet_gldm_wavelet-HHL-SmallDependenceLowGrayLevelEmphasis + -0.211209223 * normalize_glszm_ZonePercentage + -0.234543771*normalize_firstorder_Maximum + 0.6998787
